# OMRT-2. Liquid biopsy for patient stratification and monitoring of dacomitinib clinical trial in patients with EGFR amplified recurrent glioblastoma

**DOI:** 10.1093/noajnl/vdab070.028

**Published:** 2021-07-05

**Authors:** Anudeep Yekula, Robert Kitchen, Sudipto Chakrabortty, Bob Carter, Xandra Breakefield, Johan Skog, Leonora Balaj

**Affiliations:** 1 Massachusetts General Hospital, Boston, MA, USA; 2 Exosome Diagnostics, Waltham, MA, USA

## Abstract

**Introduction:**

Liquid biopsy for the detection and monitoring of brain tumors is of significant clinical interest. The ability to non-invasively profile tumors can avoid a risky biopsy and opens avenues for testing novel therapies by accurately stratifying patients to receive the right therapy. Here, we provide evidence of EV RNA-based diagnosis, patient stratification, and assessment of response to therapy in the setting of a clinical trial evaluating the efficacy of dacomitinib, an EGFR tyrosine kinase inhibitor in patients with recurrent, EGFR amplified GBM(NCT01112527).

**Methods:**

We performed RNASeq on long RNA extracted from the serum samples, pre-treatment and 1-month post-treatment.

**Results:**

Firstly, longRNASeq allowed the detection of thousands of mRNA, lincRNAs and antisense RNAs enabling the study of a wider repertoire of potential RNA based biomarkers.

Secondly, we observed a differential expression profile in serum EV RNA of GBM patients and healthy controls. Combining our findings with TCGA data and literature screening, we generated a 25 gene signature representative of critical pathways in several hallmarks of cancer.

Thirdly, we observed a differential expression profile in serum EV RNA of responders to dacomitinib compared to non-responders in pre-treatment serum. Specifically, the EV mRNAs ZNF35 and LAMTOR2 distinguish responders from non-responders (p-adjusted = 2.6E-8 and 2.4E-6, respectively) allowing potential patient stratification.

Finally, we observed a differential expression profile in serum EV RNA of responders to dacomitinib compared to non-responders in post-treatment serum. EV mRNA DNMT3A is significantly enriched (p-adjusted = 1.8E-4) in post-treatment serum of responders compared to non-responders to dacomitinib allowing potential monitoring of response to therapy.

**Conclusion:**

This study represents the first longitudinal profiling of the EV transcriptome in a cohort of genomically selected GBM patients. These findings are a tantalizing step toward liquid biopsy-based biomarkers for the detection of GBM, as well as patient stratification and monitoring.

